# Patient-specific prostate segmentation in kilovoltage images for radiation therapy intrafraction monitoring via deep learning

**DOI:** 10.1038/s43856-025-00935-2

**Published:** 2025-06-03

**Authors:** Adam Mylonas, Zeyao Li, Marco Mueller, Jeremy T. Booth, Ryan Brown, Mark Gardner, Andrew Kneebone, Thomas Eade, Paul J. Keall, Doan Trang Nguyen

**Affiliations:** 1 Image X Institute, Faculty of Medicine and Health, The University of Sydney, Sydney, NSW Australia; 2 Institute of Medical Physics, School of Physics, The University of Sydney, Sydney, NSW Australia; 3 Northern Sydney Cancer Centre, Royal North Shore Hospital, St Leonards, NSW Australia

**Keywords:** Radiotherapy, Prostate cancer

## Abstract

**Background:**

During radiation therapy, the natural movement of organs can lead to underdosing the cancer and overdosing the healthy tissue, compromising treatment efficacy. Real-time image-guided adaptive radiation therapy can track the tumour and account for the motion. Typically, fiducial markers are implanted as a surrogate for the tumour position due to the low radiographic contrast of soft tissues in kilovoltage (kV) images. A segmentation approach that does not require markers would eliminate the costs, delays, and risks associated with marker implantation.

**Methods:**

We trained patient-specific conditional Generative Adversarial Networks for prostate segmentation in kV images. The networks were trained using synthetic kV images generated from each patient’s own imaging and planning data, which are available prior to the commencement of treatment. We validated the networks on two treatment fractions from 30 patients using multi-centre data from two clinical trials.

**Results:**

Here, we present a large-scale proof-of-principle study of *x*-ray-based markerless prostate segmentation for globally available cancer therapy systems. Our results demonstrate the feasibility of a deep learning approach using kV images to track prostate motion across the entire treatment arc for 30 patients with prostate cancer. The mean absolute deviation is 1.4 and 1.6 mm in the anterior–posterior/lateral and superior–inferior directions, respectively.

**Conclusions:**

Markerless segmentation via deep learning may enable real-time image guidance on conventional cancer therapy systems without requiring implanted markers or additional hardware, thereby expanding access to real-time adaptive radiation therapy.

## Plain language summary

During radiation therapy for prostate cancer, the natural movement of the prostate can reduce treatment accuracy and compromise treatment outcomes. To track this motion, small gold markers can be surgically implanted in the prostate, but this adds cost, complexity, and potential risks. Here, we developed artificial intelligence models that can track the prostate directly from standard *x*-ray images—without the need for implanted markers or specialised equipment. We validated the models on data from 30 patients and demonstrated the feasibility of monitoring prostate motion during treatment. This approach could potentially enable real-time image guidance on existing treatment systems, helping to improve the accuracy and precision of radiation therapy.

## Abstract (web summary)

Mylonas et al. demonstrate the feasibility of using deep learning for markerless prostate segmentation in kilovoltage *x*-ray images during radiation therapy. This proof-of-principle retrospective study, involving 30 patient treatments, highlights the potential for real-time image guidance without implanted markers or additional hardware.

## Introduction

Image guidance is vital for interventional procedures, such as radiation therapy, needle biopsy and surgery. During radiation treatments, the tumour and surrounding anatomy are dynamically moving, in accordance with normal physiological processes. This makes image guidance a necessity throughout the duration of treatment to monitor tumour motion and, therefore, ensure adequate dose coverage of the tumour. Motion monitoring is essential for high-dose treatments, such as stereotactic body radiation therapy (SBRT), where high radiation dose per treatment fraction is prescribed with small geometric margins, ultimately demanding high precision^1,2^. For prostate cancer, the effect of motion can result in up to 19% less radiation dose delivered to the prostate in one fraction compared to the prescribed dose per fraction^3^. Hewson et al. found that SBRT prostate cancer patients treated without real-time motion adaptation resulted in underdosing to the clinical treatment volume (CTV) of up to −5.6% and overdosing to the rectum and bladder of up to 1.2 and 8.5%, respectively^1^. With mounting evidence on the detrimental effects of underdosing tumours and overdosing organs at risk during treatment, the American Society for Radiation Oncology recommended imaging during treatment to continuously monitor the tumour motion for high-dose radiation treatments^4^.

Real-time image-guided adaptive radiation therapy (IGART) can be used to estimate the tumour location during radiation therapy to improve dose coverage and reduce the radiation dose to healthy tissue^5^. IGART can be performed by acquiring kilovoltage (kV) images during treatment using the on-board imager that is routinely installed on conventional radiation therapy treatment systems. A robust segmentation method can be used to accurately determine the tumour position. For conventional therapy systems, real-time motion monitoring methods typically track implanted fiducial markers as surrogates to the tumour, especially for organs and tumours with low radiographic contrast, such as the prostate^6–11^. Fiducial markers and the implantation procedure adds time delays, additional costs, and risks. The treatment delays are a result of surgery wait time and the time for the markers to stabilise^12^. Some risks associated with the implantation of markers include infection, haematuria, rectal bleeding, and patient discomfort from the surgery^13,14^. Furthermore, marker migration can result in tracking errors^15^, and the metal artefacts produced from markers in computed tomography (CT) images may result in treatment image matching errors^16^. Currently, patients who are not candidates for marker implantation due to contraindications cannot receive real-time IGART. A worldwide survey relating to respiratory motion management found that 71% of radiation therapy centres wish to implement targeted radiation therapy but are limited by resources and capacity^17^. Ideally, real-time motion monitoring should not require any additional procedures or hardware. A markerless-based approach using a conventional therapy system would help make real-time IGART accessible to all patients without additional hardware.

During treatment, the treatment beam rotates around the patient, delivering radiation from multiple angles. It is important to track the prostate continuously to account for motion that may result in suboptimal tumour control and increased toxicities. Although random motion within the planning treatment volume (PTV) is accounted for in the prescribed dose, large motion can have considerable dosimetric impact. For prostate radiation therapy, the MIRAGE trial^18^ demonstrated reduced toxicities when using magnetic resonance imaging (MRI) guidance, while the SPARK trial^19^ achieved dose improvements using *x*-ray guidance. In the SPARK trial, the prostate CTV received a dose 5% less than the planned dose in 11% of treatments without real-time tracking, compared to 0% with real-time tracking^19^. In a Memorial Sloan Kettering trial, the prostate CTV received a dose 5% less than the planned dose in 6% of patients without real-time tracking, compared to 0% with real-time tracking^20^. Furthermore, motion management is critical for the coverage of smaller targets or organs at risk (such as focal boosting^21^ or urethral sparing), which are more susceptible to motion.

In this study, we perform markerless prostate segmentation in kV images with a patient-specific model by leveraging deep learning. Recently, artificial intelligence (AI) for fluoroscopic and MRI guided radiation therapy has shown great potential^22^. Markerless-based approaches have been developed for lung^23–25^, diaphragm^26^, liver^24^, pancreas^27,28^, prostate^29^, and head and neck^30^. We take an important step towards clinical implementation through a large-scale proof-of-principle of *x*-ray-based markerless tracking for globally available cancer therapy systems. Our method uses a deep learning model trained on synthetic two-dimensional (2D) images derived from the three-dimensional (3D) planning CT and prostate contour. Our study presents the full offline method development that can be incorporated into the treatment workflow.

Here, we use a patient-specific conditional Generative Adversarial Network (cGAN) to segment the prostate in 2D kV images. A patient-specific model is advantageous as it requires less data than training a generalised model. Furthermore, it allows the model to learn features most relevant to the specific patient under treatment and can be applicable to patients imaged using different imaging systems. This patient-specific approach can eliminate potential biases that may be present in generalised models^31^. Our approach leverages the patient’s own imaging and planning data that are available prior to the commencement of their treatment. The cGAN model was evaluated on two datasets using imaging data with and without markers from four different clinical sites in Australia. The datasets each have different uncertainties related to the ground truth creation. As such, we assessed the model’s performance on both datasets to gauge its effectiveness considering these varying uncertainties. Our results indicate that the prostate can be segmented without markers in *x*-ray images with a high degree of accuracy.

## Methods

### Conditional GAN framework

**Fig. 1:**
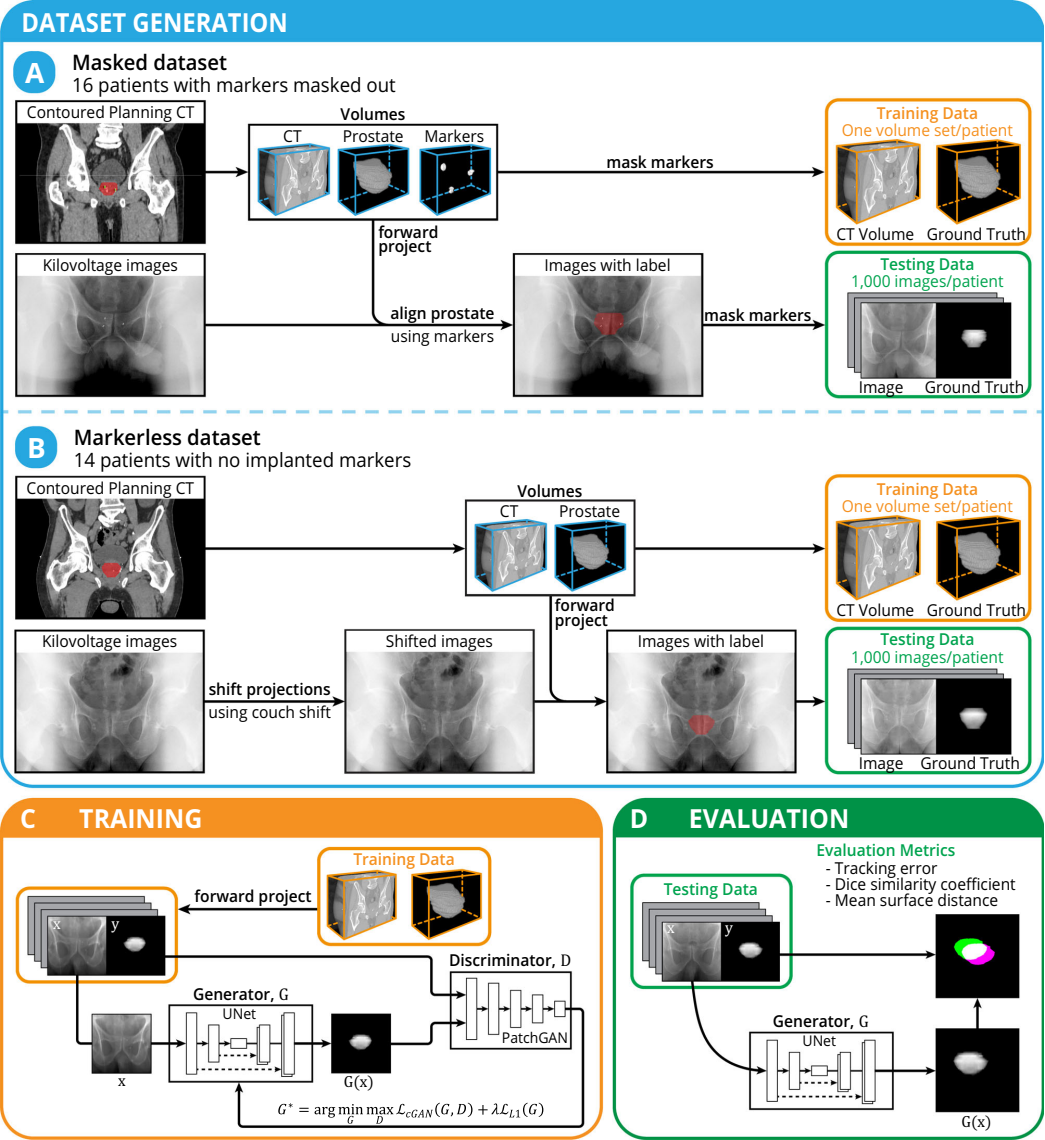
Overview of the methodology. **A** The masked dataset was generated from imaging data of prostate cancer patients with implanted fiducial markers. The markers were used to align the ground truth contour and were masked out to avoid biasing the model. **B** The markerless dataset was generated from imaging data of prostate cancer patients with no implanted fiducial markers. The kilovoltage images were shifted based on the soft tissue registration for each fraction. **C** The data were used to train a conditional Generative Adversarial Network (cGAN) for each patient consisting of a UNet generator network (*G*) and a PatchGAN discriminator network (*D*). **D** The cGAN model was evaluated using the testing data and the performance was quantified using the centroid tracking error, Dice similarity coefficient, and mean surface distance.

The tracking system uses a cGAN model for segmentation of the prostate. The training of the model involves adversarial learning between the generator network, $G,\; {{{\rm{and}}}}\; {{{\rm{the}}}}\; {{{\rm{discriminator}}}}\; {{{\rm{network}}}},\,D.\; {{{\rm{The}}}}\; {{{\rm{cGAN}}}}\; {{{\rm{model}}}}\; {{{\rm{is}}}}\; {{{\rm{trained}}}}\; {{{\rm{to}}}}\; {{{\rm{replicate}}}}\; {{{\rm{a}}}}\; {{{\rm{prostate}}}}\; {{{\rm{segmentation}}}}\; {{{\rm{given}}}}\; {{{\rm{a}}}}\; {{{\rm{pelvis}}}}\; {{{\rm{kV}}}}\; {{{\rm{image}}}}\; {{{\rm{as}}}}\; {{{\rm{input}}}}.\;{{{\rm{The}}}}\; {{{\rm{generator}}}},\,G,\;{{{\rm{takes}}}}\; {{{\rm{the}}}}\; {{{\rm{input}}}}\; {{{\rm{kV}}}}\; {{{\rm{image}}}},\,x,\;{{{\rm{and}}}}\; {{{\rm{creates}}}}\; {{{\rm{a}}}}\; {{{\rm{segmentation}}}}\; {{{\rm{image}}}}\; G(x).\;{{{\rm{The}}}}\; {{{\rm{discriminator}}}},\,D,\; {{{\rm{classifies}}}}\; {{{\rm{whether}}}}\; {{{\rm{the}}}}\; {{{\rm{paired}}}}\; {{{\rm{image}}}},\,{xy}$, comes from the training set or the generator network as shown in Fig. 1C. The cGAN was initialised with a normal distribution and trained to minimise the loss function:

1 $${G}^{ * }={{\arg }}\mathop{\min }_{G}\mathop{\max }_{D}{{{{\mathcal{L}}}}}_{{cGAN}}\left(G,D\right)+\lambda {{{{\mathcal{L}}}}}_{L1}\left(G\right)$$

where λ is a constant (set to 100 for this implementation) and:

2 $${{{{\mathcal{L}}}}}_{{{{\rm{cGAN}}}}}\left(G,D\right)={E}_{x,y}\left[\log D\left(x,y\right)\right]+{E}_{x}\left[\log \left(1-{{{\rm{D}}}}\left({{{\rm{x}}}},{{{\rm{G}}}}\left({{{\rm{x}}}}\right)\right)\right)\right]$$

3 $${{{{\mathcal{L}}}}}_{L1}(G)={{E}_{x,y}\|y-G\left(x\right)\|}_{1}$$

The cGAN implementation used for this study was based on the Pix2pix model^32^. A UNet was used for the generator architecture, and a PatchGAN for the discriminator architecture. The detailed network architectures are shown in Supplementary Fig. 1. A patient-specific model was trained for each patient using 36,000 digitally reconstructed radiographs (DRRs) per patient. The DRRs were generated using the Reconstruction Toolkit^33^ and the Insight Toolkit^34^, using the traditional DRR algorithm as derived by Madden et al. ^35^. The DRRs were produced using two volumetric images: the planning CT and prostate contour volume. Each volume was forward-projected to produce a DRR at every degree over a full 360° rotation, resulting in a set of 360 images.

Prior to training, data augmentation for each patient was performed 100 times by randomly shifting the CT geometry up to 10 mm and rotating up to 10° and then computing a new set of DRRs to replicate possible treatment setup error and anatomical motion^36^. In total, 36,000 DRRs were created for each patient with a size of 512 × 512 pixels. The images produced from the planning CT were each paired with the respective image produced from the prostate contour. Each model was trained for 20 epochs with a batch size of four and a learning rate of 0.0002 using the Adam optimiser. The models were trained on a desktop computer with an Intel® Xeon® Gold 6248 R processor (3.0 GHz) with 256 GB RAM and a NVIDIA® RTX A6000 GPU.

### Clinical application

**Fig. 2:**
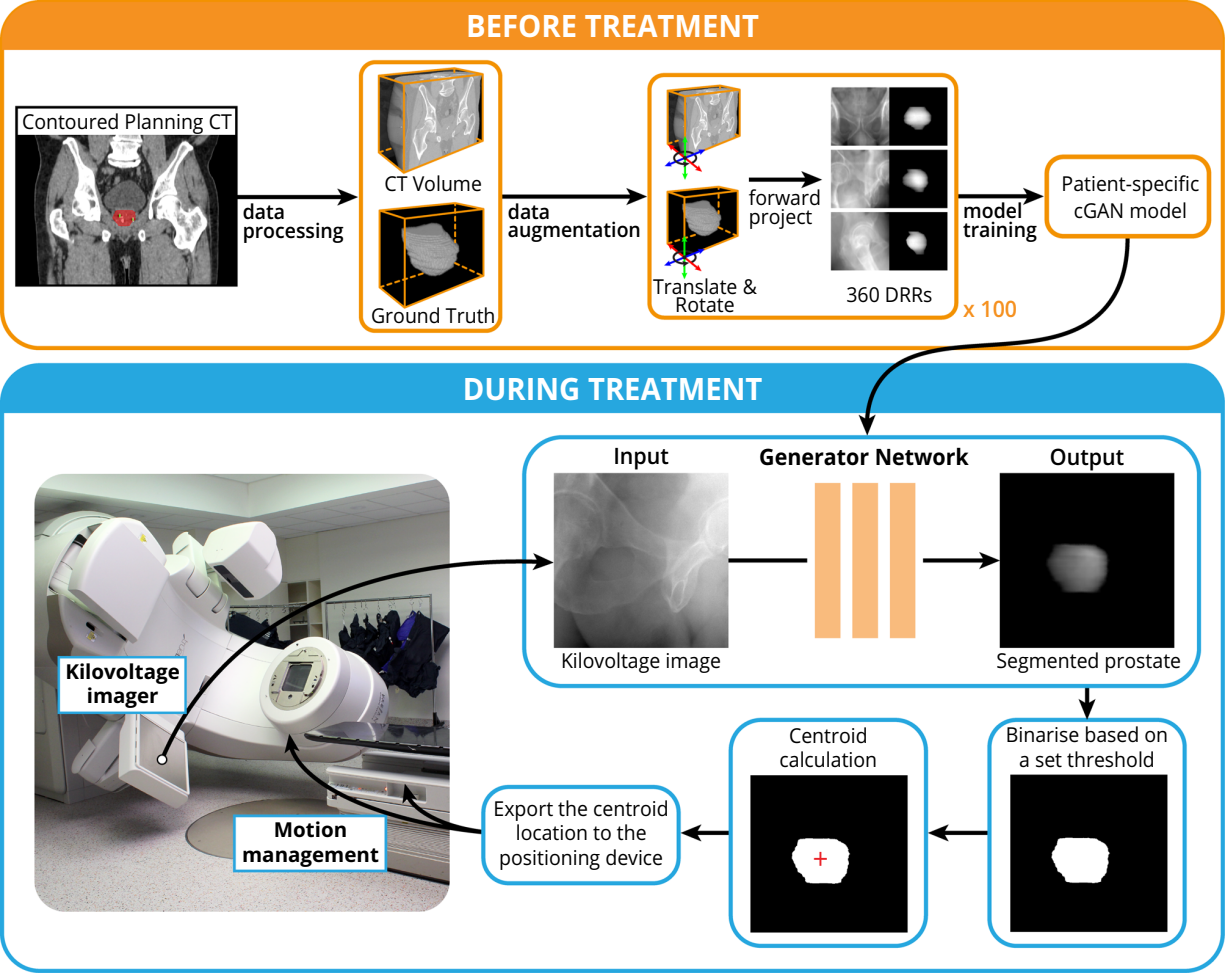
Simulated real-time clinical study of the deep learning method. The automatic prostate tracking workflow is divided into two main phases: before treatment and during treatment. A patient-specific conditional generative adversarial network (cGAN) is trained prior to the patient’s treatment using 36,000 synthetic 2D images derived from the 3D planning CT and prostate contour data. The cGAN generator network is used during the treatment to segment the prostate. The location of the segmented prostate can potentially be used for motion management. DRR, digitally reconstructed radiographs.

The cGAN model can be incorporated into the treatment workflow for intrafraction monitoring of the prostate in kV images (Fig. 2). For clinical implementation, the central component of the workflow is the generator network for prostate segmentation. The generator network of the cGAN produces a prediction image (prostate segmentation) based on a kV input image. The prediction image is binarised using a threshold value (10% in this implementation) to give the final segmentation, and the centroid of the segmentation is calculated. If multiple unconnected regions are detected, the centroid of the largest region is calculated. The calculated centroid location can be exported to the positioning systems to allow for real-time motion adjustments during the treatment.

### Masked dataset

The masked dataset was generated using imaging data of patients with implanted fiducial markers, which were masked out for training and analysis (Fig. 1A). The dataset was constructed using the imaging data of 16 prostate cancer patients undergoing radiation therapy in the TROG 15.01 SPARK clinical trial^37^ (10.25910/qg5d-6058^38^). The SPARK clinical trial was approved by Hunter New England Local Health District Human Research Ethics Committee (Reference No: 15/06/17/3.01; ClinicalTrials.gov Identifier: NCT02397317). Participants provided informed consent to participate in the SPARK clinical trial. The requirement for additional consent for the use of de-identified data in unspecified future research was waived by the Committee on 22 March 2021, under which this study is covered. The patients that were used in this study were treated on the TrueBeam (Varian Medical Systems, Palo Alto, CA, USA) linear accelerator (linac) across three different sites (Calvary Mater Newcastle, Australia; Westmead Hospital, Australia; Peter MacCallum Cancer Centre, Australia). We collected the planning CT, physician contours, and kV images from two fractions of each patient associated with this cohort. The kV images were acquired during patient setup to reconstruct the cone beam computed tomography (CBCT). We utilised 500 kV images from each fraction, giving a total of 16,000 kV images. Each patient had three cylindrical gold fiducial markers implanted in their prostate.

The training data were the 3D planning CT and 3D prostate contour, while the test data were generated using the kV images from two fractions. The prostate contour volume was projected as a 2D DRR for each kV image angle. The ground truth was generated by aligning the prostate-only DRR with the kV images based on the implanted fiducial markers. The marker positions were labelled using a previously developed deep learning framework^10^ with manual verification. Following alignment, the fiducial markers were masked out in all volumes, DRRs, and kV images to avoid biasing the model. The masking algorithm uses the *regionfill* function in MATLAB (R2021b, MathWorks, Natick, MA, USA) to smoothly interpolate inwards from the pixel values surrounding the marker by calculating the discrete Laplacian and solving the Dirichlet boundary value problem. Poisson noise was applied to the kV images following interpolation.

### Markerless dataset

The markerless dataset was generated using imaging data of patients with no implanted fiducial markers (Fig. 1B). The dataset was constructed using the imaging data of 14 prostate cancer patients undergoing radiation therapy in the OPTIMAL clinical trial^39^. The OPTIMAL clinical trial was approved by Northern Sydney Local Health District Human Research Ethics Committee (Reference No: RESP/17/344; ClinicalTrials.gov Identifier: NCT03386045). Participants provided informed consent for the clinical trial, including the secondary objective of evaluating the potential to develop markerless tracking technology utilising intrafraction monitoring. This trial treated patients on the TrueBeam STx (Varian Medical Systems, Palo Alto) Linac at Royal North Shore Hospital, Australia. We collected the planning CT, physician contours, and kV images from two fractions associated with this cohort. As with the masked dataset, the kV images were acquired during patient setup to reconstruct the CBCT with 500 images from each fraction, giving a total of 14,000 kV images. The training data were generated in the same way as the masked dataset. Since these patients did not have implanted fiducial markers, the test data ground truth was generated using shifts based on the image registration performed between the planning CT and treatment CBCT. The 2D kV images were shifted based on the 3D couch shift using Eq. 4:

4 $$\widehat{{{{\bf{P}}}}}\left(\widehat{{{{\bf{M}}}}}\left(t\right)|\theta \right)=\left(\begin{array}{c}\widehat{{{{\boldsymbol{u}}}}}\\ \widehat{{{{\boldsymbol{v}}}}}\end{array}\right)=\frac{{SID}}{{SAD}-\left(\widehat{{{{\boldsymbol{x}}}}}\cdot \cos \theta +\widehat{{{{\boldsymbol{z}}}}}\cdot \sin \theta \right)}\left(\begin{array}{c}\widehat{{{{\boldsymbol{x}}}}}\cdot \sin \theta -\widehat{{{{\boldsymbol{z}}}}}\cdot \cos \theta \\ \widehat{{{{\boldsymbol{y}}}}}\end{array}\right)$$

where SID is the linac source-isocentre distance, SAD is the linac source-aperture distance, ($\hat{x},\hat{y},\hat{z}$) are the 3D shifts, and ($\hat{u},\hat{v}$) are the resulting 2D shifts.

After shifting the kV images, prostate-only DRRs were generated for each kV image angle. Therefore, the ground truth in the markerless dataset is defined by the average location of the prostate rather than the real-time location.

### Statistics and reproducibility

The models were tested on the unseen kV images of 30 patients to evaluate the accuracy of the prostate segmentation and the tracking system. For each patient, kV images from two fractions were used, giving 1000 test images per patient (500 per fraction). For the masked dataset, ground truth generation involved manual verification of 48,000 marker positions to ensure high-quality data. Due to the time-consuming nature, we prioritised evaluating variability between patients and limited the analysis to two fractions per patient to include more patients. For the markerless dataset, only two fractions of data were available for each patient. The cGAN segmentation was binarised using a 10% threshold and was compared to the ground truth segmentation for the analysis.

The generator’s ability to be used in an automated tracking system was evaluated by comparing the centroid of the segmentations. The tracking system error was defined as the cGAN segmentation centroid minus the ground truth segmentation centroid, calculated in the anterior–posterior/lateral (AP/LAT) and superior–inferior (SI) directions. The errors were reported at the patient coordinate system using the source-isocentre distance/source-detector distance ratio ( = 1.5) as the correction factor giving a pixel size of 0.26 mm. The generator’s ability to produce accurate prostate segmentations was evaluated for each patient model. The performance was quantified by calculating the Dice similarity coefficient (DSC) and mean surface distance (MSD) between the cGAN segmentation and the ground truth.

The centroid errors, DSC, and MSD were quantified by calculating the mean ± standard deviation, as well as the 5 and 95th percentiles. Additionally, the mean absolute deviation (MAD) of the centroid error was calculated. The correlation between the observed motion and centroid error in the masked dataset was evaluated using the Pearson correlation coefficient and Bland–Altman analysis. We also investigated the relationship between the quantitative results and the kV imager angle. All quantitative analyses were performed using MATLAB (R2021b, MathWorks, Natick, MA, USA) and GraphPad Prism (version 10.2.3, GraphPad Software, Boston, MA, USA).

### Reporting summary

Further information on research design is available in the Nature Portfolio Reporting Summary linked to this article.

## Results

### Conditional GAN training and retrospective validation

Figure 1 provides a detailed overview of our deep learning study. For our study, two separate datasets were used to evaluate the performance of the cGAN segmentation: the masked dataset (Fig. 1A) and the markerless dataset (Fig. 1B). Ground truth annotation by clinicians in each kV image was not feasible due to the low soft tissue contrast of the images. Therefore, the ground truth was generated using a different approach for each dataset. The masked dataset was generated from imaging data of 16 prostate cancer patients with implanted fiducial markers. The markers were used to annotate the real-time location of the prostate in the kV images. During testing of the model, the real-time prediction of the prostate location could be compared with the ground-truth prostate location, using the implanted markers as surrogates. The fiducial markers were removed in the training and testing data to avoid biasing the deep learning model. Marker removal was achieved by smoothly interpolating inwards from the pixel values surrounding the marker, with Poisson noise subsequently applied to the kV images. Manual visual inspection of each kV image was performed to ensure that the markers were masked and no longer visible.

The markerless dataset included imaging data of 14 prostate cancer patients with no implanted fiducial markers. A rigid shift of the kV images was applied based on an expert’s registration performed between the planning CT and reconstructed CBCT. Therefore, the ground truth in the markerless dataset was defined by the average location of the prostate rather than the real-time location. The markerless dataset does not provide a gold standard for intrafraction motion, as the ground truth is based on the average location. The observed motion may be a result of the prostate not being at the average location rather than the detection of intrafraction motion.

For this approach to be clinically feasible, the patient-specific model must be trained using data available prior to the patient’s first treatment. In the conventional clinical workflow, a patient will receive a planning CT several days or weeks prior to the first treatment, which is used by clinicians to contour the relevant volumes and plan the treatment. Therefore, we can use this available data to train the model with sufficient time prior to the patient’s first treatment. The inputs to the model were the planning CT and prostate contour 3D volumes (Fig. 1C). The volumes were forward-projected to produce 36,000 synthetic 2D images simulating kV images from different projection angles. The images produced from the planning CT were each paired with the respective image produced from the prostate contour. To segment the prostate in the 2D kV images, we used a cGAN with a UNet for the generator architecture and a PatchGAN for the discriminator architecture. The detailed network architecture is shown in Supplementary Fig. 1. On the hardware described in the Methods, the mean ( ± standard deviation) training time, including data generation and augmentation, was 9.7 ± 0.7 h with a maximum time of 10.7 h.

### Tracking performance

**Fig. 3:**
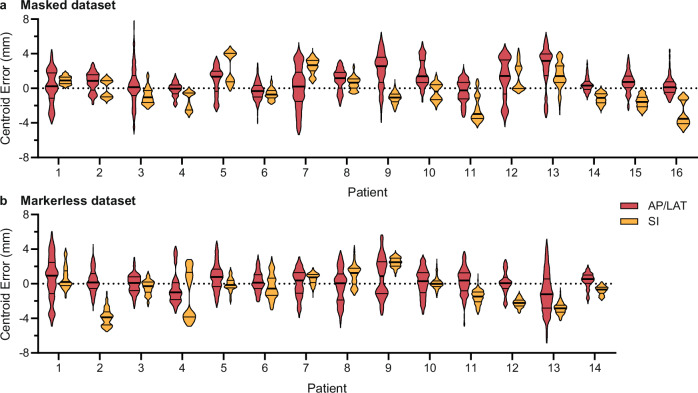
Violin plots showing centroid error distributions. Centroid errors of the conditional Generative Adversarial Network segmentation compared to the ground truth, shown for each patient in the anterior–posterior/lateral (AP/LAT, red) and superior–inferior (SI, yellow) directions for the masked dataset (**a**) and the markerless dataset (**b**). Each violin plot represents data from *n* = 1000 images, with the bars indicating the median (thick) and quartiles (thin).

We evaluated the models using kV images from two fractions of each patient’s treatment (Fig. 1D). The kV images were acquired during patient setup to reconstruct the CBCT. The centroid errors of the cGAN segmentations for all patients are represented in Fig. 3 and Supplementary Tables 1 and 2. The centroid errors are presented in the AP/LAT and SI directions with a pixel size of 0.26 mm. The AP and LAT directions are combined due to the rotational geometry of the treatment beam. For the masked dataset, the mean ( ± standard deviation) error across all patients was 0.7 ± 1.9 mm and −0.2 ± 1.9 mm in the AP/LAT and SI directions, respectively (Supplementary Table 1). The MAD was 1.4 and 1.5 mm in the AP/LAT and SI directions, respectively. The 5 and 95th percentiles were −2.4 and 4.0 mm in the AP/LAT direction, and −3.4 and 3.5 mm in the SI direction (Supplementary Table 1). Similar performance was observed for the markerless dataset. The mean error for all patients was 0.1 ± 1.8 mm and −0.6 ± 1.9 mm in the AP/LAT and SI directions, respectively (Supplementary Table 2). The MAD was 1.4 and 1.6 mm in the AP/LAT and SI directions, respectively. The 5 and 95th percentiles were −2.8 and 3.1 mm in the AP/LAT direction, and −4.0 and 2.6 mm in the SI direction (Supplementary Table 2).

**Fig. 4:**
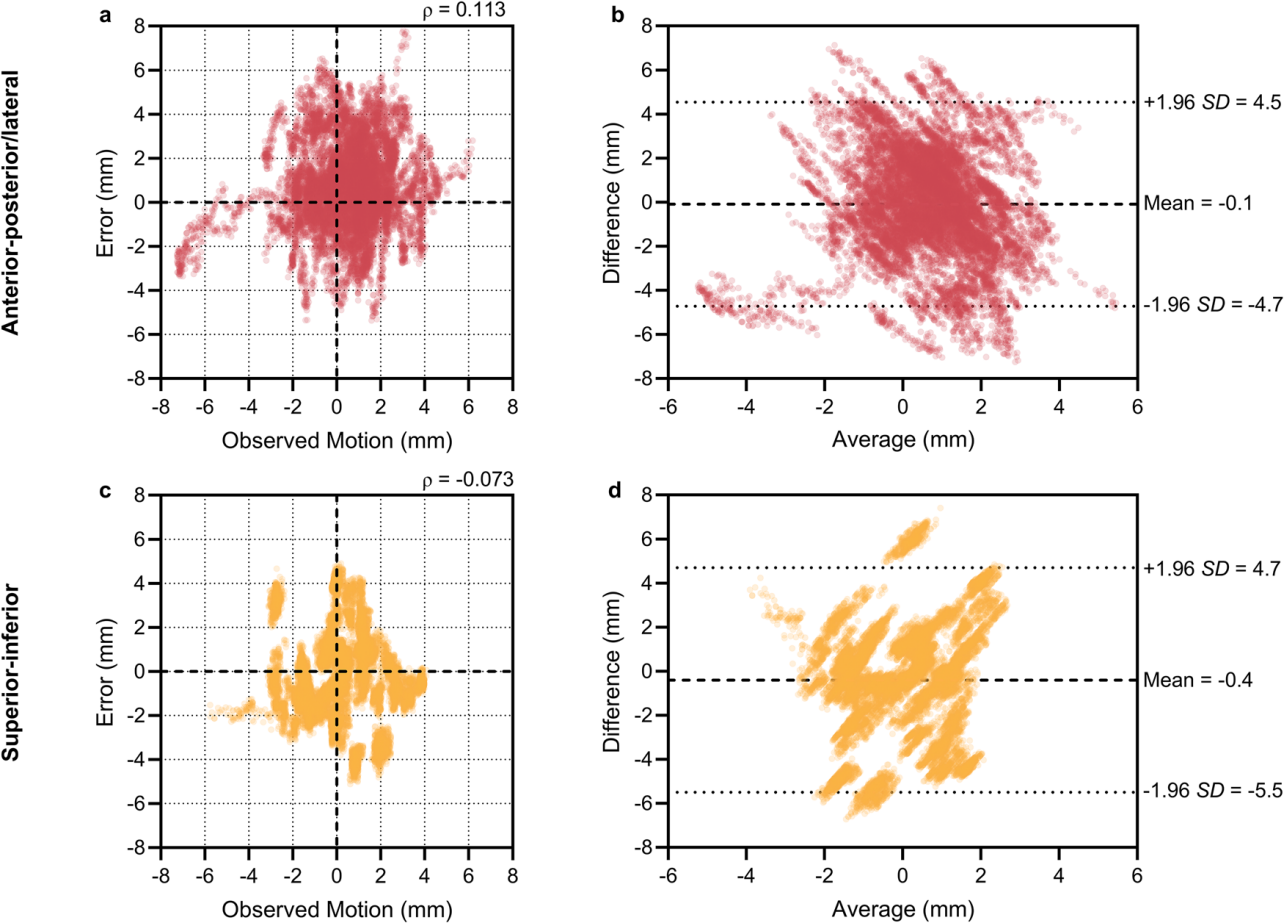
Comparison of observed motion and centroid error in the masked dataset. Scatter plots (**a, c**) and Bland–Altman plots (**b, d**) comparing the observed motion and centroid error for each direction (*n* = 16,000). The top row (**a, b**) corresponds to the anterior–posterior/lateral direction, and the bottom row (**c, d**) to the superior–inferior direction. The Pearson correlation coefficient is shown in (**a, c**).

The correlation between the magnitude of motion and the absolute errors for all patients in the masked dataset is shown in Fig. 4. The error was found to be independent of the observed motion with low Pearson correlation coefficients of 0.113 and −0.073 in the AP/LAT and SI directions, respectively (Fig. 4a, c). Figure 4 shows the Bland–Altman plot of the observed motion versus the centroid error. The mean is low in both directions, suggesting minimal bias between the measurements, and the difference between the measurements does not tend to get larger as the average increases.

### Segmentation performance

**Fig. 5:**
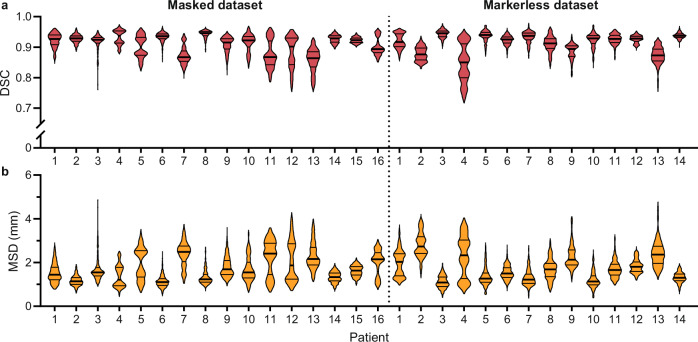
Violin plots of geometric assessment metrics. Dice similarity coefficient (DSC; **a**) and mean surface distance (MSD; **b**) between the conditional Generative Adversarial Network segmentation and the ground truth for each patient. Each violin plot represents data from *n* = 1000 images, with the bars indicating the median (thick) and quartiles (thin).

The performance of the cGAN in terms of the DSC and MSD is presented in Fig. 5 and Supplementary Tables 3 and 4. For the masked dataset, the predicted and ground truth segmentations have high agreement, with a mean ( ± standard deviation) DSC across all patients of 0.91 ± 0.04. The 5 and 95th percentiles of the DSC were 0.84 and 0.95. The mean MSD was 1.7 ± 0.7 mm, with 5 and 95th percentiles of 0.9 and 3.0 mm (Supplementary Table 3). Similar performance was observed for the markerless dataset with a mean DSC for all patients of 0.91 ± 0.04 and 5 and 95th percentiles of 0.84 and 0.95. The mean MSD was 1.8 ± 0.7 mm, with 5 and 95th percentiles of 0.9 and 3.1 mm (Supplementary Table 4).

**Fig. 6:**
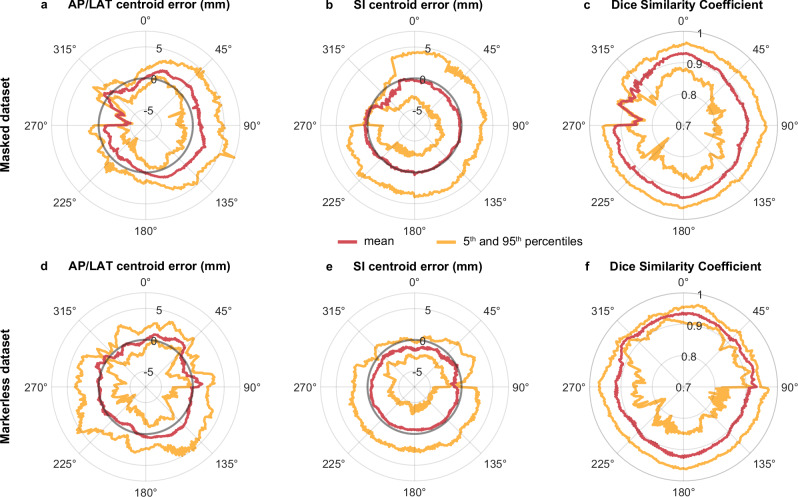
Centroid error and Dice similarity coefficient (DSC) based on imager angle. Mean (red) and 5^th^ and 95^th^ percentiles (yellow) for centroid error in the anterior–posterior/lateral (AP/LAT) direction (**a, d**), centroid error in the superior–inferior (SI) direction (**b, e**), and DSC (**c, f**) across the radiation therapy treatment arc. The top row (**a–c**) corresponds to the masked dataset (*n* = 16,000) and the bottom row (**d–f**) corresponds to the markerless dataset (*n* = 14,000).

The mean centroid error and DSC results at each imager angle are presented in Fig. 6. For the masked dataset, the mean error in the SI direction was consistent across all angles while the error in the AP/LAT direction has a positive offset for the first 180° (Fig. 6a). For the masked dataset, the mean error in the AP/LAT and SI direction was consistent across all angles (Fig. 6b). The DSC results show consistently worse performance for each dataset at the post-oblique angles (Fig. 6a, b). At these angles, the *x*-ray path length through the patient is towards the maximum as it beams through the pelvis. The difference in the model performance in the range of 270–330° for the masked dataset and 290–90° for the markerless dataset is a result of the variable number of samples at each angle. The kV images were obtained through a sub-arc acquisition method. Consequently, there are fewer samples available for angles within the aforementioned ranges compared to other segments of the arc.

**Fig. 7:**
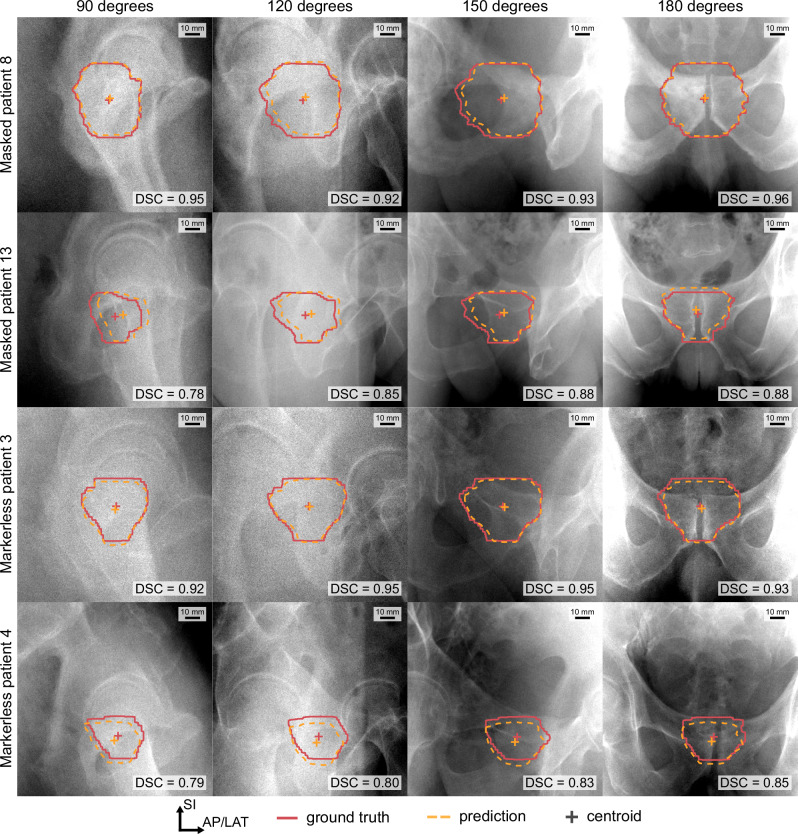
Predicted segmentations and ground truth of four patients. Comparison of the conditional generative adversarial network segmentation (dashed yellow) and ground truth (solid red) at imager angles of 90°, 120°, 150°, and 180°. The best and worst performing patient in each dataset, based on the mean Dice similarity coefficient (DSC), are shown. The DSC is reported for each image. AP/LAT, anterior–posterior/lateral; SI, superior–inferior.

The time taken for the trained network to generate the segmentation was ∼10 ms per image using the hardware described in the Methods. An example of the cGAN and ground truth segmentations at different image angles of four patients are shown in Fig. 7. This figure demonstrates the best and worst performing patient in each dataset based on the mean DSC (Fig. 7). It can be observed that for all patients, there is strong agreement in the shape of the segmentation. While there is strong agreement in shape, there is an offset in the centroid positions for the worse-performing segmentations.

**Fig. 8:**
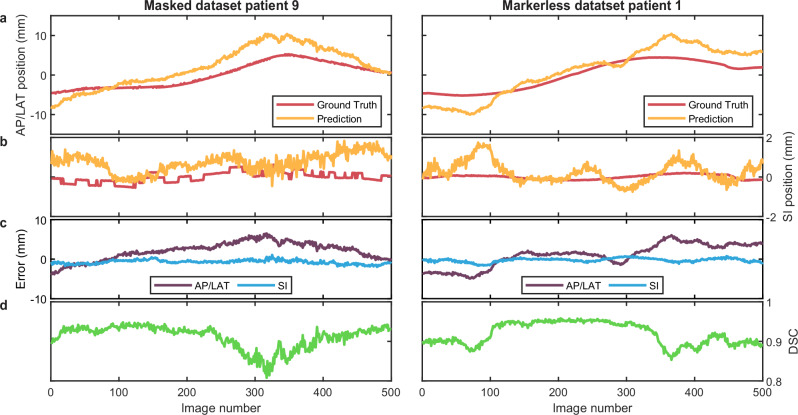
Tracking and Dice similarity coefficient (DSC) results for two patients. **a, b** The prostate centroid position in the anterior–posterior/lateral (AP/LAT) direction (**a**) and superior–inferior (SI) direction (**b**) of the conditional Generative Adversarial Network (cGAN) segmentation compared to the ground truth. **c** Centroid error between the cGAN segmentation and the ground truth. **d** DSC between the cGAN segmentation and the ground truth.

The results presented in Fig. 8 show example tracking results for the average performing patient in each dataset based on the mean DSC. One fraction each with 500 kV images are shown. The tracking system was able to maintain tracking throughout both fractions. The error in the SI direction is typically lower compared to the AP/LAT direction (Fig. 8c). The predicted and ground truth segmentations agree well with each other with a DSC around 0.9 across both patients (Fig. 8d). Videos of the tracking for both patients are shown in Supplementary Movies 1 and 2.

## Discussion

Our results suggest that a patient-specific deep learning model has the potential for motion management using real-time markerless prostate segmentation. We accomplished this using data acquired from a conventional radiation therapy system at four different cancer treatment centres. The tracking accuracy was 0.7 ± 1.9 mm and −0.2 ± 1.9 mm in the AP/LAT and SI directions for the masked dataset. A similar performance was observed for the markerless dataset with 0.1 ± 1.8 mm and −0.6 ± 1.9 mm in the AP/LAT and SI directions, respectively. The masked dataset results indicate that the method maintains consistent accuracy across varying magnitudes of motion, as the errors are uncorrelated with the observed motion (Pearson correlation coefficients: 0.113 and −0.073 for AP/LAT and SI directions, respectively). The Bland–Altman analysis further supports this, showing minimal bias between the measurements and no trend of larger errors with increasing averages. However, there were many instances where the error exceeded the motion. Adjusting table position based on this signal would unlikely result in an overall reduction of uncertainty.

From a study of 17 patients, Langen et al. reported that the 3D prostate displacement was >3 mm and >5 mm approximately 14 and 3% of the time, respectively^40^. These prostate motion measurements are similar to those reported by Su et al. indicating 17 and 5% of time for the corresponding displacements^41^. Both studies highlighted the increased likelihood of displacement of the prostate with elapsed time^40,41^. Therefore, real-time motion monitoring is essential for treatment regimens where the treatment time is increased, such as with hypofractionated SBRT. For radiation therapy treatments, a PTV margin is applied around the prostate to account for treatment setup errors and intrafraction motion. For prostate radiation therapy, a PTV of 10 mm is used when using skin marks or bony anatomy for set up and 5–8 mm when using soft tissue registration or implanted markers^42^. These margins were further reduced for motion management treatments. In the SPARK trial, which used *x*-ray guidance, margins of 3 mm posteriorly and 5 mm in other directions were used^19^. In the MIRAGE trial, which used MRI guidance, 2 mm isotropic margins were used^18^.

Our results are insensitive to motion in the plane perpendicular to the detector plane, as the model estimates the position in the 2D kV image frame of reference. For clinical use, there will need to be implementation of an algorithm to infer the 3D target coordinates from the 2D kV images. For marker-based tumour tracking, a method for 3D target estimation using the marker positions in 2D images has been clinically implemented^43^. Other successful approaches include using a 3D Gaussian PDF^44^, Bayesian inference^45,46^, or a Kalman filter^47^. These estimation methods could be adapted using the segmentation boundary or centroid for our approach. While our accuracy is reported in 2D, making direct comparisons with other studies difficult, we can expect that the model would be useful for detecting high-motion cases. Given the high mean DSC achieved on both datasets (masked: 0.91 ± 0.04; markerless: 0.91 ± 0.04), gating could be performed when a defined percentage of prostate moves outside of a set treatment boundary.

Current *x*-ray-based methods for tracking the prostate during radiation therapy rely on implanted fiducial markers^6–11^. While some marker-based approaches achieved sub-millimetre accuracy, our markerless approach nevertheless achieves millimetre accuracy. There is minimal literature on tracking the prostate without markers. Zhao et al. ^29^ developed a deep learning model to identify the rectangular bounding box containing the prostate in simulated images for ten patients at three specific angles: AP, left–lateral, and oblique^29^. Zhao et al. achieved a MAD of 1.58–1.67 mm across the three angles when tested on simulated images. Our large multi-centre study demonstrated comparable performance on treatment-acquired kV images with a MAD of 1.4–1.6 mm across all angles and datasets. However, a larger error distribution was observed in our study. The larger error distribution may be attributed to the more challenging evaluation on treatment-acquired kV images with scatter presence, compared to evaluation on simulated images at defined angles. Zhao et al. reported an increase in the MAD to 2.29 mm when evaluating the models on kV images for a subset of patients demonstrating the increased difficulty. Deep learning approaches have been developed for other sites including lung^23–25^, diaphragm^26^, liver^24^, pancreas^27,28^, and head and neck^30^ for guided radiation therapy. Our method performs with a similar accuracy when compared to the markerless approaches for other anatomical sites. However, it should be noted that markerless tracking of the prostate and pancreas is more difficult than the other sites due to the lower soft tissue contrast. MRI linacs are an alternative for IGART due to improved soft tissue contrast compared to kV images^12^. However, MRI-linacs are an expensive treatment option for patients and are not widely available compared to standard linacs^48^. Deep learning approaches for markerless MRI-guided radiation therapy have typically focused only on lung^49,50^, and liver^51^ tracking.

Our model has several features that make it an ideal candidate for clinical implementation. First, the model takes 9.7 ± 0.7 h on average to train including data generation and augmentation, making it feasible for patient-specific training in between the patient’s planning session and the first treatment. Multiple models can be trained simultaneously, and the training time could be further optimised through computer enhancements. Second, the inference time of the model was 10 ms on average per image. This makes the model suitable for real-time applications, as the AAPM Task Group 264 defines real-time as a system latency below 500 ms^52^. A single model approach for tracking at all angles across the entire treatment arc is less computationally intensive and more clinically relevant than using several models for discrete angles. Third, the model produces a segmentation of the prostate, which when combined with 3D target estimation can be beneficial for other applications such as real-time dose optimisation^53^. Finally, the model is patient-specific, allowing it to learn features relevant to the specific patient and imaging system. The robustness of health AI algorithms is a major concern^54^. Often, the performance of an algorithm can be correlated to the particular data used for training. However, this is not a concern for our study as the patient-specific model has been tested across four different cancer treatment centres, achieving a similar performance across all patients. As the model is agnostic to the prostate characteristics, it could be easily adapted to include other treatment targets such as the pelvic lymph nodes or organs at risk.

However, there are risks associated with tumour tracking algorithms. If the algorithm produces inaccurate results, it could potentially disrupt a treatment that is proceeding as expected. One strategy to address this would be to incorporate a confidence metric so that low-confidence results do not disrupt the treatment. Additionally, the beam delivery could be paused only when the tumour motion exceeds a pre-set tolerance for a pre-defined time to negate random errors. Other risks can include failure to detect clinically significant motion. One potential solution would be to incorporate redundancy through multiple tracking algorithms. The additional algorithms tracking the surrounding organs at risk may assist with detecting all significant motion.

Our study is not without limitations. The first limitation is the uncertainties related to the ground truth of the prostate in the kV images. Due to the low soft tissue contrast in the kV images, it is not possible to manually contour or label the prostate. Therefore, other solutions were required to generate the ground truth. We developed two methods of producing the ground truth. The aim was that results together, from both methods, would reduce the uncertainties related to the results. The masked dataset was generated from imaging data of prostate cancer patients with implanted fiducial markers. The markers were used to annotate the real-time location of the prostate in the kV images and were then masked out. The method for localisation of the markers has shown sub-millimetre accuracy^10^. However, fiducial markers are subject to 1–2 mm surrogacy errors^55–57^ and may therefore limit the accuracy of the ground truth prostate segmentation.

The markerless dataset was generated from imaging data with no implanted fiducial markers. Since it is not possible to annotate the real-time location, the kV images were rigidly shifted based on image registration performed between the planning CT and treatment CBCT giving an average location. The uncertainties related to soft-tissue registration between the CBCT and planning CT are typically between 1–3 mm^58,59^. The results from these two ground truths combined provide increased certainty of the model performance for markerless tracking. The two approaches rely on the planning CT prostate contour, which can have uncertainties relating to intraobserver and interobserver variability. Additionally, utilising the planning CT contour for training does not account for potential deformation of the prostate between planning and treatment. Prostate deformation has been reported to have a mean and standard deviation of 0.6 and 1.7 mm, respectively^60^. Furthermore, it was found that AP displacement of the prostate centre of gravity is highly correlated with deformation in the middle-anterior and posterior segments^60^. Therefore, the prostate deformation and resulting shift in the centre of gravity may affect the model performance.

The augmentation applied to the training dataset only accounted for the motion of the entire patient, excluding anatomical motion. In previous work, we used realistic deformations to train a deep learning model for head and neck tumour segmentation, demonstrating that this approach enhanced robustness to patient motion^30^. However, head and neck tumours exhibit different motion and deformation characteristics compared to the prostate. Initial experiments using a similar augmentation approach to shift the prostate and deform surrounding soft tissue did not improve performance and substantially increased training time due to deformation computations. Therefore, we focused on optimising performance and training time by using rigid shifts alone.

The second limitation is that the model was evaluated using kV images acquired during patient setup rather than intrafraction kV images. For the masked dataset, the intrafraction kV images were collimated. As a result, the surrounding anatomy is not visible, making the images unsuitable for segmentation. Additionally, for the markerless dataset, we prioritised the quality of our ground truth and hence used kV images from patient setup for this study. To generate the ground truth for the markerless dataset, image registration was required between the treatment 3D CBCT and planning CT to determine the average location. Therefore, we could not generate the ground truth for the intrafraction kV images in the markerless dataset. While kV images from patient setup provide superior quality to intrafraction kV images, state-of-the-art clinical systems provide solutions to minimise the effect of megavoltage (MV) scatter from the treatment beam and provide an improved kV image quality. One such solution is trigged imaging that is incorporated into Varian systems^61^. Triggered imaging improves kV image quality by placing the treatment beam on hold prior to acquisition of each triggered image in order to eliminate the effect of MV scatter. The simple solution of frame averaging has been previously used to reduce noise in the images^62^. As our model is trained on a case-by-case basis, it can benefit from future research that improves kV image quality.

To the best of our knowledge, our study is the first large-scale proof-of-principle of *x*-ray-based markerless tracking for globally available cancer therapy systems, providing an important step towards clinical implementation. The results demonstrate the potential of our method to be expanded to other soft tissue organs, such as the pancreas, liver, and kidneys. Our approach only requires *x*-ray images during treatment, which in principle, covers all linear accelerators from Elekta, Varian, and other manufacturers. Our markerless method will help make IGART treatments accessible for all patients, eliminating the time delays, costs and risk associated with marker implantation. Future work will look at the development of a single software solution with a model for clinical implementation. The software solution can then be experimentally tested on a conventional radiation therapy system using a phantom and prospectively with a quality assurance procedure^63^.

## Supplementary information

Supplementary Information

Description of Additional Supplementary Files

Supplementary Data 1

Supplementary Movie 1

Supplementary Movie 2

Reporting Summary

## Data Availability

The SPARK clinical trial dataset (10.25910/qg5d-6058)^38^, used in this study for the masked dataset, is available under a Creative Commons Attribution 4.0 Licence. The OPTIMAL clinical trial dataset, used in this study for the markerless dataset, is not currently available to protect participant privacy while the trial is ongoing. Thomas Eade (thomas.eade@health.nsw.gov.au) can be contacted for more information about the OPTIMAL clinical trial dataset. The data points plotted in Figs. 3–6 and 8 can be found in Supplementary Data 1.

## Code availability

The conditional GAN used in this study is based on the Pix2pix model (https://github.com/junyanz/pytorch-CycleGAN-and-pix2pix)^32^. The forward projections used for training were generated with the Reconstruction Toolkit (https://www.openrtk.org/)^33^.
