# Supplementary material for: Patient-specific prostate segmentation in kilovoltage images for radiation therapy intrafraction monitoring via deep learning
Source: Commun Med (Lond). 2025 Jun 3;5:212. doi: 10.1038/s43856-025-00935-2 (PMC12134301; doi:10.1038/s43856-025-00935-2)
Supplement: Supplementary file 1 — Supplementary Information [file 43856_2025_935_MOESM1_ESM.pdf]

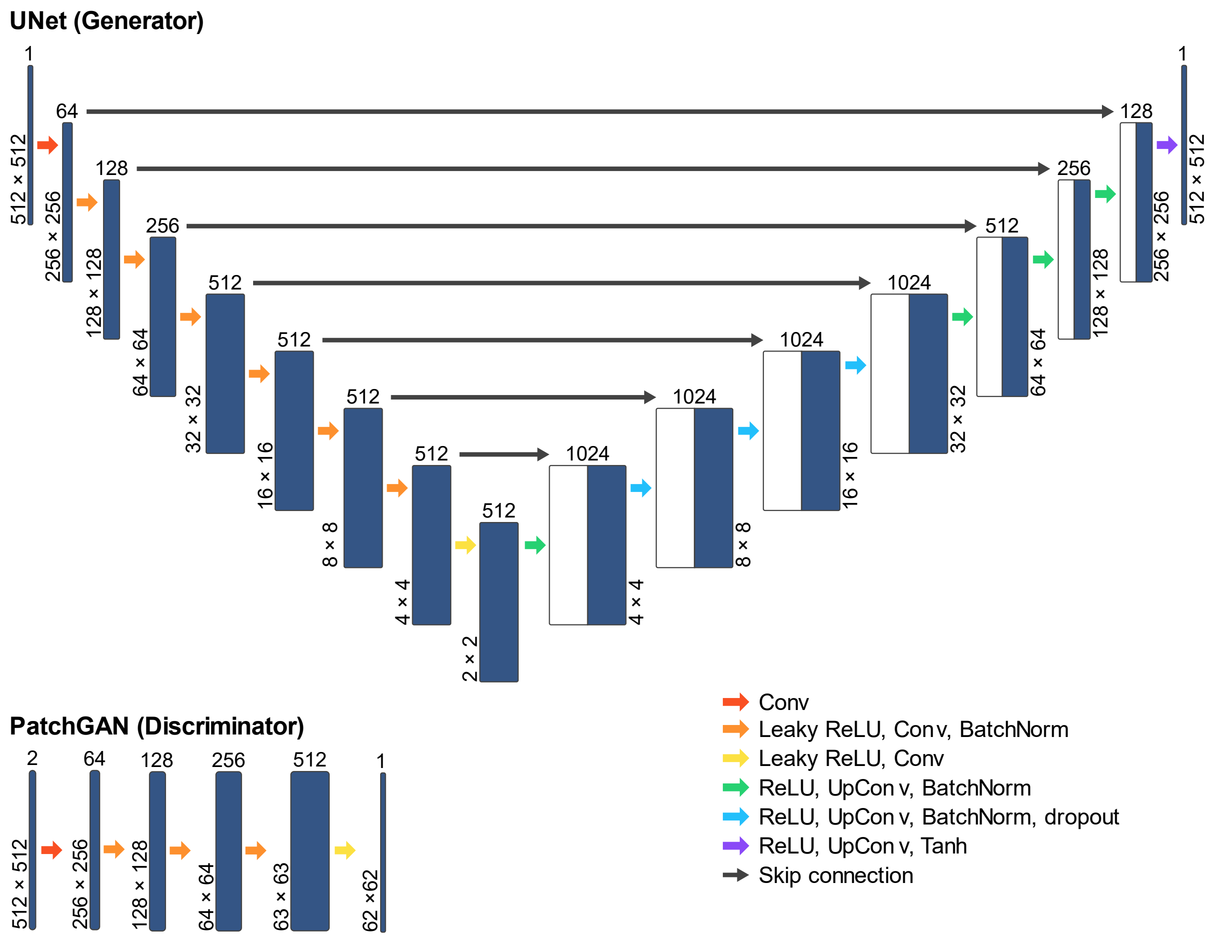

Supplementary Fig. 1 | UNet (Generator) and PatchGAN (Discriminator) network architectures. The generator network takes a kilovoltage image as input and produces a prostate segmentation. The discriminator takes 70 × 70 patches of the input image to determine if the segmentations are real or fake. Conv, convolution; ReLU, rectified linear unit; BatchNorm, batch normalisation; UpConv, upconvolution.

Supplementary Table 1 | Centroid errors for individual patients in the masked dataset. For each patient in the masked dataset, the mean ± standard deviation (SD), 5th percentile, and 95th percentile of the centroid errors between the conditional Generative Adversarial Network segmentation and the ground truth are reported. The errors are calculated in the anterior-posterior/lateral (AP/LAT) and superior-inferior (SI) directions.

Patient	AP/LAT Centroid Error (mm)			SI Centroid Error (mm)		
	**Mean ± SD**	**5th percentile**	**95th percentile**	**Mean ± SD**	**5th percentile**	**95th percentile**
1	0.2 ± 1.9	-3.2	3.1	0.9 ± 0.5	0.3	1.7
2	0.7 ± 1.1	-1.1	2.4	0.0 ± 1.0	-1.3	1.3
3	0.3 ± 2.3	-3.4	4.6	-0.9 ± 0.9	-2.1	1.2
4	-0.1 ± 0.8	-1.7	1.1	-1.5 ± 1.0	-2.9	-0.2
5	0.9 ± 1.6	-2.1	3.0	2.4 ± 1.7	0.1	4.4
6	-0.3 ± 1.1	-2.0	1.5	-0.7 ± 0.6	-1.6	0.5
7	0.1 ± 2.4	-4.2	3.7	2.5 ± 0.9	1.0	3.8
8	1.0 ± 1.2	-1.3	2.8	0.6 ± 0.7	-0.5	1.8
9	2.1 ± 2.1	-1.5	5.1	-1.1 ± 0.6	-2.3	-0.1
10	1.8 ± 1.6	-0.5	4.4	-0.4 ± 1.0	-1.8	1.0
11	-0.3 ± 1.3	-2.8	1.5	-2.4 ± 1.4	-4.0	0.5
12	1.1 ± 2.4	-3.0	4.1	1.4 ± 1.5	-0.3	3.9
13	2.4 ± 2.4	-2.5	5.3	1.5 ± 1.5	-1.2	3.8
14	0.4 ± 0.8	-0.7	2.1	-1.2 ± 0.6	-2.2	-0.2
15	0.7 ± 1.2	-1.3	2.8	-1.6 ± 0.7	-2.7	-0.4
16	0.3 ± 1.1	-1.2	2.5	-3.0 ± 1.4	-4.6	-0.8
Overall	0.7 ± 1.9	-2.4	4.0	-0.2 ± 1.9	-3.4	3.5

Supplementary Table 2 | Centroid errors for individual patients in the markerless dataset. For each patient in the markerless dataset, the mean ± standard deviation (SD), 5th percentile, and 95th percentile of the centroid errors between the conditional Generative Adversarial Network segmentation and the ground truth are reported. The errors are calculated in the anterior-posterior/lateral (AP/LAT) and superior-inferior (SI) directions.

Patient	AP/LAT Centroid Error (mm)			SI Centroid Error (mm)		
	Mean ± SD	5th percentile	95th percentile	Mean ± SD	5th percentile	95th percentile
1	0.6 ± 2.5	-3.7	4.6	0.7 ± 1.3	-0.8	3.4
2	0.3 ± 1.4	-1.8	2.7	-3.9 ± 1.0	-5.2	-2.0
3	0.0 ± 1.1	-1.8	1.9	-0.4 ± 0.8	-1.8	0.9
4	-0.5 ± 1.8	-2.6	3.5	-1.3 ± 2.7	-4.6	2.5
5	0.8 ± 1.6	-1.8	3.7	-0.1 ± 0.8	-1.5	1.3
6	0.2 ± 1.2	-1.6	2.3	-0.3 ± 1.4	-2.4	2.1
7	0.1 ± 1.6	-2.9	2.3	0.6 ± 0.6	-0.5	1.4
8	-0.2 ± 2.0	-3.4	2.8	1.0 ± 1.2	-0.9	3.0
9	0.8 ± 2.3	-2.7	4.5	2.5 ± 0.6	1.5	3.4
10	0.1 ± 1.6	-2.6	2.6	0.1 ± 0.7	-0.9	1.5
11	0.2 ± 1.5	-2.2	2.9	-1.6 ± 0.8	-3.0	-0.5
12	0.1 ± 1.3	-2.3	2.3	-2.2 ± 0.5	-3.1	-1.4
13	-1.0 ± 2.3	-4.3	3.2	-2.8 ± 0.6	-3.9	-1.8
14	0.4 ± 0.9	-1.6	1.6	-0.8 ± 0.5	-1.7	0.0
Overall	0.1 ± 1.8	-2.8	3.1	-0.6 ± 1.9	-4.0	2.6

Supplementary Table 3 | Geometric assessment metrics for individual patients in the masked dataset. The mean ± standard deviation (SD), 5th percentile, and 95th percentile of the Dice similarity coefficient (DSC) and mean surface distance (MSD) between the conditional Generative Adversarial Network segmentation and the ground truth are reported for each patient in the masked dataset.

Patient	DSC			MSD (mm)		
	**Mean ± SD**	**5th percentile**	**95th percentile**	**Mean ± SD**	**5th percentile**	**95th percentile**
1	0.92 ± 0.02	0.87	0.95	1.5 ± 0.4	0.9	2.4
2	0.93 ± 0.01	0.90	0.95	1.2 ± 0.2	0.8	1.6
3	0.91 ± 0.03	0.85	0.94	1.7 ± 0.6	1.1	3.0
4	0.93 ± 0.02	0.89	0.96	1.4 ± 0.5	0.8	2.3
5	0.90 ± 0.04	0.84	0.95	2.0 ± 0.7	0.9	3.0
6	0.93 ± 0.02	0.91	0.95	1.1 ± 0.3	0.8	1.6
7	0.87 ± 0.03	0.83	0.93	2.4 ± 0.6	1.4	3.3
8	0.94 ± 0.01	0.92	0.96	1.3 ± 0.3	1.0	2.0
9	0.91 ± 0.03	0.86	0.94	1.8 ± 0.5	1.2	2.6
10	0.91 ± 0.03	0.86	0.95	1.7 ± 0.6	0.9	2.7
11	0.88 ± 0.05	0.81	0.95	2.2 ± 0.8	0.9	3.3
12	0.88 ± 0.05	0.79	0.95	2.1 ± 0.9	0.9	3.6
13	0.86 ± 0.04	0.78	0.91	2.3 ± 0.6	1.4	3.5
14	0.93 ± 0.01	0.90	0.95	1.3 ± 0.2	0.9	1.7
15	0.92 ± 0.01	0.91	0.94	1.6 ± 0.2	1.2	2.0
16	0.90 ± 0.03	0.86	0.95	2.0 ± 0.6	1.0	2.8
Overall	0.91 ± 0.04	0.84	0.95	1.7 ± 0.7	0.9	3.0

Supplementary Table 4 | Geometric assessment metrics for individual patients in the markerless dataset. The mean ± standard deviation (SD), 5th percentile, and 95th percentile of the Dice similarity coefficient (DSC) and mean surface distance (MSD) between the conditional Generative Adversarial Network segmentation and the ground truth are reported for each patient in the markerless dataset.

Patient	DSC			MSD (mm)		
	**Mean ± SD**	**5th percentile**	**95th percentile**	**Mean ± SD**	**5th percentile**	**95th percentile**
1	0.92 ± 0.02	0.88	0.95	2.0 ± 0.6	1.2	2.9
2	0.88 ± 0.02	0.84	0.91	2.8 ± 0.6	1.9	3.8
3	0.94 ± 0.01	0.92	0.96	1.1 ± 0.3	0.7	1.6
4	0.85 ± 0.06	0.75	0.94	2.3 ± 0.9	0.9	3.7
5	0.94 ± 0.02	0.90	0.96	1.4 ± 0.4	0.8	2.3
6	0.92 ± 0.02	0.90	0.95	1.5 ± 0.3	1.1	2.1
7	0.93 ± 0.02	0.89	0.96	1.3 ± 0.4	0.8	2.1
8	0.91 ± 0.03	0.86	0.95	1.7 ± 0.5	0.9	2.5
9	0.89 ± 0.03	0.83	0.92	2.3 ± 0.6	1.6	3.4
10	0.92 ± 0.02	0.88	0.95	1.2 ± 0.4	0.7	1.9
11	0.93 ± 0.02	0.90	0.95	1.7 ± 0.4	1.2	2.4
12	0.93 ± 0.01	0.90	0.94	1.8 ± 0.3	1.4	2.4
13	0.87 ± 0.03	0.82	0.92	2.4 ± 0.6	1.6	3.4
14	0.94 ± 0.01	0.92	0.95	1.3 ± 0.2	0.9	1.7
Overall	0.91 ± 0.04	0.84	0.95	1.8 ± 0.7	0.9	3.1
