# Supplementary material for: Patient-specific prostate segmentation in kilovoltage images for radiation therapy intrafraction monitoring via deep learning
Source: Commun Med (Lond). 2025 Jun 3;5:212. doi: 10.1038/s43856-025-00935-2 (PMC12134301; doi:10.1038/s43856-025-00935-2)
Supplement: Supplementary file 2 — Description of Additional Supplementary Files [file 43856_2025_935_MOESM2_ESM.pdf]

Description of Additional Supplementary Files

File name: Supplementary Data 1.

Description: Spreadsheet of the data used to generate Figs. 3–6 and 8, with a description of the corresponding data for each figure provided in the Summary sheet.

File name: Supplementary Movie 1.

Description: Prostate segmentation using the conditional Generative Adversarial Network (dashed yellow) compared to the ground truth (solid red) for Patient 9 in the masked dataset, shown across two treatment fractions.

File name: Supplementary Movie 2.

Description: Prostate segmentation using the conditional Generative Adversarial Network (dashed yellow) compared to the ground truth (solid red) for Patient 1 in the markerless dataset, shown across two treatment fractions.
