# Supplementary material for: Patient-specific prostate segmentation in kilovoltage images for radiation therapy intrafraction monitoring via deep learning
Source: Commun Med (Lond). 2025 Jun 3;5:212. doi: 10.1038/s43856-025-00935-2 (PMC12134301; doi:10.1038/s43856-025-00935-2)
Supplement: Supplementary file 6 — Reporting Summary [file 43856_2025_935_MOESM6_ESM.pdf]

nature portfolio

Corresponding author(s): Adam Mylonas

Last updated by author(s): May 27, 2025

Reporting Summary

Nature Portfolio wishes to improve the reproducibility of the work that we publish. This form provides structure for consistency and transparency in reporting. For further information on Nature Portfolio policies, see our Editorial Policies and the Editorial Policy Checklist.

Statistics

For all statistical analyses, confirm that the following items are present in the figure legend, table legend, main text, or Methods section.

n/a	Confirmed	
☐	☒	The exact sample size (*n*) for each experimental group/condition, given as a discrete number and unit of measurement
☒	☐	A statement on whether measurements were taken from distinct samples or whether the same sample was measured repeatedly
☒	☐	The statistical test(s) used AND whether they are one- or two-sided<br>*Only common tests should be described solely by name; describe more complex techniques in the Methods section.*
☒	☐	A description of all covariates tested
☒	☐	A description of any assumptions or corrections, such as tests of normality and adjustment for multiple comparisons
☐	☒	A full description of the statistical parameters including central tendency (e.g. means) or other basic estimates (e.g. regression coefficient) AND variation (e.g. standard deviation) or associated estimates of uncertainty (e.g. confidence intervals)
☒	☐	For null hypothesis testing, the test statistic (e.g. *F*, *t*, *r*) with confidence intervals, effect sizes, degrees of freedom and *P* value noted<br>*Give P values as exact values whenever suitable.*
☒	☐	For Bayesian analysis, information on the choice of priors and Markov chain Monte Carlo settings
☒	☐	For hierarchical and complex designs, identification of the appropriate level for tests and full reporting of outcomes
☐	☒	Estimates of effect sizes (e.g. Cohen's *d*, Pearson's *r*), indicating how they were calculated

Our web collection on statistics for biologists contains articles on many of the points above.

Software and code

Policy information about availability of computer code

Data collection	The conditional Generative Adversarial Network used in this study is based on the Pix2pix model (https://github.com/junyanz/pytorch-CycleGAN-and-pix2pix). The model was implemented using Python 3.10.9, PyTorch 2.0.0, and CUDA 12.1. The forward projections used for training were generated with the Reconstruction Toolkit (https://www.openrtk.org/).
Data analysis	Data analyses and plotting were performed using MATLAB R2021a, except for violin, scatter, and Bland–Altman plots, which were generated using GraphPad Prism 10.

For manuscripts utilizing custom algorithms or software that are central to the research but not yet described in published literature, software must be made available to editors and reviewers. We strongly encourage code deposition in a community repository (e.g. GitHub). See the Nature Portfolio guidelines for submitting code & software for further information.

Data

Policy information about availability of data

All manuscripts must include a data availability statement. This statement should provide the following information, where applicable:

- Accession codes, unique identifiers, or web links for publicly available datasets
- A description of any restrictions on data availability
- For clinical datasets or third party data, please ensure that the statement adheres to our policy

The SPARK clinical trial dataset (https://doi.org/10.25910/qg5d-6058), used in this study for the masked dataset, is available under a Creative Commons Attribution

4.0 Licence.

The OPTIMAL clinical trial dataset, used in this study for the markerless dataset, is not currently available to protect participant privacy while the trial is ongoing. Thomas Eade (thomas.eade@health.nsw.gov.au) can be contacted for more information about the OPTIMAL clinical trial dataset.

The data points plotted in Figs. 3–6 and 8 can be found in Supplementary Data 1.

Human research participants

Policy information about studies involving human research participants and Sex and Gender in Research.

Reporting on sex and gender	As this was a prostate cancer study, only male participants were included.
Population characteristics	In the SPARK clinical trial, participants were aged 18 years or older and had histologically proven prostate adenocarcinoma. Participants had low or intermediate-risk disease, based on PSA levels, Gleason grade, and stage, with an Eastern Cooperative Oncology Group (ECOG) performance status of 0-2. Participants were suitable for definitive external beam radiotherapy and had the ability to have three gold fiducial markers placed in the prostate.<br><br>For the Optimal clinical trial, participants were required to have histologically proven prostate adenocarcinoma, with all levels of PSA eligible. Participants also had an ECOG performance status of 0-2 and were suitable for high-dose irradiation to the prostate.
Recruitment	This was a retrospective study that utilised data from patients recruited in the SPARK and Optimal clinical trials
Ethics oversight	SPARK was approved by the Hunter New England Local Health District HREC (Reference No: 15/06/17/3.01). Participants provided informed consent to participate in the SPARK clinical trial. The requirement for additional consent for the use of de-identified data in unspecified future research was waived by the Committee on 22 March 2021, under which this study is covered.<br><br>OPTIMAL was approved by the Northern Sydney Local Health District HREC (Reference No: RESP/17/344). Participants provided informed consent for the study, including the secondary objective of evaluating the potential to develop markerless tracking technology utilising intrafraction monitoring.

Note that full information on the approval of the study protocol must also be provided in the manuscript.

Field-specific reporting

Please select the one below that is the best fit for your research. If you are not sure, read the appropriate sections before making your selection.

☒ Life sciences ☐ Behavioural & social sciences ☐ Ecological, evolutionary & environmental sciences

For a reference copy of the document with all sections, see nature.com/documents/nr-reporting-summary-flat.pdf

Life sciences study design

All studies must disclose on these points even when the disclosure is negative.

Sample size	The method was applied to data from 16 patients in the SPARK clinical trial and 14 patients in the Optimal clinical trial. Two fractions were used from each patient's treatment, with 500 images per fraction.
Data exclusions	Data were excluded if incomplete imaging datasets prevented the method from being performed. For the SPARK dataset, only data from the three centres using a Varian TrueBeam were included.
Replication	The method was replicated across a total of 30 patients, with two fractions used for each patient.
Randomization	All patients were used that had complete datatsets.
Blinding	No blinding was implemented in this study, as it was a retrospective analysis.

Reporting for specific materials, systems and methods

We require information from authors about some types of materials, experimental systems and methods used in many studies. Here, indicate whether each material, system or method listed is relevant to your study. If you are not sure if a list item applies to your research, read the appropriate section before selecting a response.

Materials & experimental systems

n/a	Involved in the study
☒	☐ Antibodies
☒	☐ Eukaryotic cell lines
☒	☐ Palaeontology and archaeology
☒	☐ Animals and other organisms
☐	☒ Clinical data
☒	☐ Dual use research of concern

Methods

n/a	Involved in the study
☒	☐ ChIP-seq
☒	☐ Flow cytometry
☒	☐ MRI-based neuroimaging

Clinical data

Policy information about clinical studies

All manuscripts should comply with the ICMJE guidelines for publication of clinical research and a completed CONSORT checklist must be included with all submissions.

Clinical trial registration	SPARK: NCT02397317<br>OPTIMAL: NCT03386045
Study protocol	The full protocols for the SPARK and OPTIMAL clinical trials can be obtained upon request. Please contact Adam Mylonas (adam.mylonas@sydney.edu.au) for the SPARK protocol and Thomas Eade (thomas.eade@health.nsw.gov.au) for the OPTIMAL protocol.
Data collection	The SPARK clinical trial dataset (https://doi.org/10.25910/qg5d-6058), used in this study for the masked dataset, is available under a Creative Commons Attribution 4.0 Licence.<br><br>Data collection for the OPTIMAL clinical trial is being conducted at Royal North Shore Hospital, Australia, with an estimated completion date in 2026.
Outcomes	This study is not related to the primary or secondary outcomes of the SPARK clinical trial and was completed using publicly available data.<br><br>This study relates to a secondary outcome of the OPTIMAL clinical trial: the potential for developing markerless tracking technology utilising intrafraction monitoring.
